# Evaluation of 6-mercaptopurine in a cell culture model of adaptable triple-negative breast cancer with metastatic potential

**DOI:** 10.18632/oncotarget.26978

**Published:** 2019-06-04

**Authors:** Balraj Singh, Vanessa N. Sarli, Hannah E. Kinne, Anna Shamsnia, Anthony Lucci

**Affiliations:** ^1^ Department of Breast Surgical Oncology, The University of Texas MD Anderson Cancer Center, Houston, TX, USA; ^2^ Morgan Welch Inflammatory Breast Cancer Research Program and Clinic, The University of Texas MD Anderson Cancer Center, Houston, TX, USA

**Keywords:** cancer evolution, metabolic adaptability in cancer, inflammatory breast cancer, minimal residual disease, TET2

## Abstract

Progenitor-like cancer cells that can survive in reversible quiescence when faced with various challenges in the body are often behind disease progression. A lack of glutamine in culture medium, which eliminates >99.9% of proliferating SUM149 triple-negative breast cancer cells, selects such adaptable, pan-resistant cells. Our data support the hypothesis that a lack of glutamine forces the selection of an epigenetic state that does not require a high level of TET2, thus selecting an “undifferentiated” therapy-resistant phenotype as seen in TET2-mutant cancers. Our data suggesting that highly adaptable cells are generated through reprograming of the epigenome and transcriptome led us to evaluate low-dose 6-mercaptopurine as a potential therapy in our model. We found that a long treatment with low-dose 6-mercaptopurine inhibited the proliferation of these adaptable cells to a greater extent than it inhibited parental cells. Importantly, a small percentage of adaptable cells survived a low-dose 6-mercaptopurine treatment in a reversible quiescence, analogous to the persistence of abnormal progenitor-like cells in inflammatory bowel disease, which stays in a durable remission with a 6-mercaptopurine treatment. Based on a biomarkers analysis, a long treatment with 6-mercaptopurine or aspirin partially reversed epithelial to mesenchymal transition in adaptable cancer cells. A cell culture model of adaptable cancer cells that persist in the body will help in discovering superior therapies that can be offered before the disease advances to metastasis.

## INTRODUCTION

Cancer progression is an evolution-like process, which is shaped by various selection pressures serving as bottlenecks [1–6]. Whether it is primary cancer, minimal residual disease (MRD) that persists after surgery and other therapies, distant metastasis, or even a cancer cell line, all comprise a large percentage of proliferative cells and a very small percentage of other cells whose role can be viewed as the preservation of cancer. While primary breast cancer is removed surgically, the presence of MRD could offer an opportunity for therapeutic intervention at a point when the disease is more treatable than metastasis [7–9]. This would require the ability to distinguish poor-prognosis MRD at high risk for metastasis from the MRD that does not lead to metastasis as well as the ability to disable poor-prognosis MRD with safe therapies. Our approach to solving this difficult problem involves realistic modeling of the cells that drive cancer evolution in the body, leading to metastasis, and evaluating the anticancer efficacy of potential therapeutic agents on such cells. The approach relies essentially on three elements: 1) choice of resistant cell lines that have a high capacity to generate cellular diversity; 2) selection of rare, highly evolvable cancer cells that survive and overcome a bottleneck in the form of a severe metabolic challenge; and 3) evaluation of potential therapeutic agents in long-term assays so as to reveal their efficacy on evolvable cells rather than proliferative cells.

While multiclonal cancer evolution in the body faces a variety of bottlenecks that enforce selection of tumor adaptability, a cancer cell line cultured in an artificially rich medium proliferates without such bottlenecks. Certain attributes, e.g., embryo-like plasticity and a capacity to generate cellular diversity, empower cancer cells to successfully adapt to and survive not just one but multiple bottlenecks, including nutrient deficiencies in the body. Our working hypothesis is that, although cell proliferation is a dominant phenotype in cell culture, rare cells in a cancer cell line are endowed with these adaptive attributes and the capacity to survive in reversible quiescence (a defining characteristic of MRD) when faced with bottlenecks. Therefore, although most (proliferating) cells in cell culture are phenotypically different than MRD, the rare cancer cells that can successfully adapt to engineered bottlenecks in cell culture may in fact be phenotypically similar to MRD. If MRD stays dormant most of the time, it may not have adverse consequences. One important characteristic of poor-prognosis MRD may be its tendency to break dormancy often while maintaining a robust component of reversible dormancy.

This study describes a strategy for testing potential therapeutic agents that may be effective against highly adaptable therapy-resistant cancer cells such as the ones present in poor-prognosis MRD in TNBC. To model deep therapeutic resistance associated with multiclonal evolution, we chose the SUM149 cell line, which was derived from a triple-negative inflammatory breast cancer that had not responded to neoadjuvant chemotherapy and radiation therapy (S. Ethier, personal communication) for in-depth functional and molecular studies. SUM149 cell line contains the mutations typically observed in advanced TNBC, e.g., those affecting TP53, PTEN, RB pathway, and a BRCA1 mutation [10]. After investigating several other bottlenecks that can be applied in cell culture, we chose glutamine deficiency for the following main reasons: 1) the rare metabolically adaptable (MA) cells (0.01% in population) that succeed in meeting this challenge have the capacity to meet other metabolic challenges such as a lack of glucose or oxygen; 2) MA cells not only can adapt to manage without exogenous glutamine (e.g., by upregulating glutamate-ammonia ligase), but they also are adaptable overall based on their embryo-like gene expression and high rate of epithelial-to-mesenchymal transition (EMT); 3) MA cells are resistant to chemotherapeutic drugs and other agents targeting cell proliferation; and 4) MA cells metastasize to multiple organs in nude mice [11–14]. A lack of the non-essential amino acid glutamine proved to be a good choice because it presents a severe challenge but rare highly adaptable cells are eventually able to overcome it. Here, we present data that explain how a lack of glutamine could force the selection and reprogramming of rare cancer cells toward a therapy-resistant state. We also describe an approach for evaluating therapeutic agents that may inhibit cancer cell adaptability. We specifically evaluated 6-mercaptopurine (6-MP), a drug that induces long-term remission in inflammatory bowel disease (IBD) [15], which could potentially interfere with the RNA processing (post-transcriptional modifications in transcriptome) that may be important in cellular adaptability.

## RESULTS

### Phenotypic selection of adaptable TNBC cells under a bottleneck

**Figure 1:**
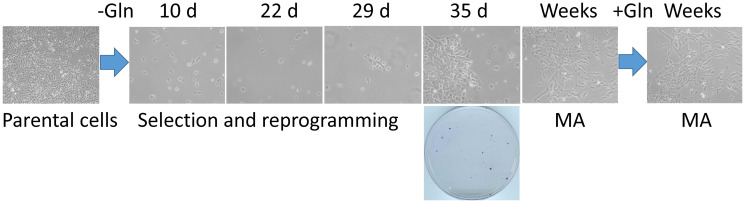
A cell culture model of the rare cancer cells that survive a severe metabolic challenge and “evolve” to emerge as highly adaptable. Triple-negative breast cancer SUM149-Luc cells were plated in 10-cm dishes (5 × 10^5^ per dish) in culture medium containing dialyzed FBS and no glutamine (Gln). While >99.9% of the cells died quickly, a small number of cells survived in quiescence for 3–4 weeks; there were innumerable abortive attempts at cell growth during this period. We postulate that a few cells in this initial period of 3–4 weeks “evolved” to a point that they eventually succeeded in forming colonies. Shown are representative cell cultures (10 × magnification) at various stages, along with a stained dish at 5 weeks (representative image taken from data in reference 13). Metabolically adaptable (MA) cancer cells selected in this manner can be cultured indefinitely in a medium without or with glutamine; representative MA cultures depicting mesenchymal morphology in both media are shown.

Our approach to a selection of highly adaptable cancer cells that can survive in reversible quiescence is described in Figure 1. SUM149 TNBC cells plated on a dish in complete medium would reach confluency in 7 days. However, it takes 5 weeks for a small number of colonies to emerge in glutamine-free medium. The long lag phase of 3–4 weeks before healthy colonies begin to develop after many cells fail in such attempts represents a crucial period during which a small percentage of cells survive and adapt to a severe and prolonged bottleneck. Although we do not know which specific permutations of genetic and epigenetic alterations allowed successful “evolution” under this bottleneck in individual cells, our gene expression and gene amplification/deletion data obtained with the MA cells (after they have been cultured for several weeks in glutamine-free medium and then in glutamine-containing medium), along with the results of our functional studies with MA cells summarized in the Introduction, are very informative in guiding our thinking about the mechanisms that enable cell survival and adaptation under a severe metabolic bottleneck [11–14]. Most of the gene expression changes in MA cells reflect the changes needed under the most difficult circumstance of trying to survive and grow without glutamine [12]. Once the bottleneck has been overcome, most of these changes (e.g., embryo-like gene expression and plasticity, high rate of EMT, overexpression of the *FTO* gene) may not be important on an ongoing basis in cell culture, particularly in proliferative cells [13].

### Low TET2 expression in SUM149-MA cells

How does a prolonged lack of glutamine that kills >99.9% of cancer cells ultimately select rare, highly adaptable cancer cells that are resistant to a variety of challenges, including therapeutic agents aimed at proliferative cells? Upon being shifted to a glutamine-free medium, most proliferative cells that are highly dependent on glutamine for their growth and redox regulation quickly die, within a day or at most few days. The rare survivor cells may use a variety of strategies, including selection of advantageous genomic and epigenetic features and possible reprogramming of the epigenome and transcriptome under these (metabolically) challenging conditions, ultimately yielding colonies of more “evolutionarily fit” resistant cancer cells [12]. A lack of glutamine could lead to a low level of α-ketoglutarate (a cofactor for many enzymes, including those affecting the epigenome and transcriptome); this is supported by findings of low glutamine levels in poorly perfused areas of tumors (or even in cancer cell lines subjected to low-glutamine medium) resulting in reduced intracellular levels of α-ketoglutarate, thus inhibiting histone demethylation and promoting dedifferentiation [16].

**Figure 2:**
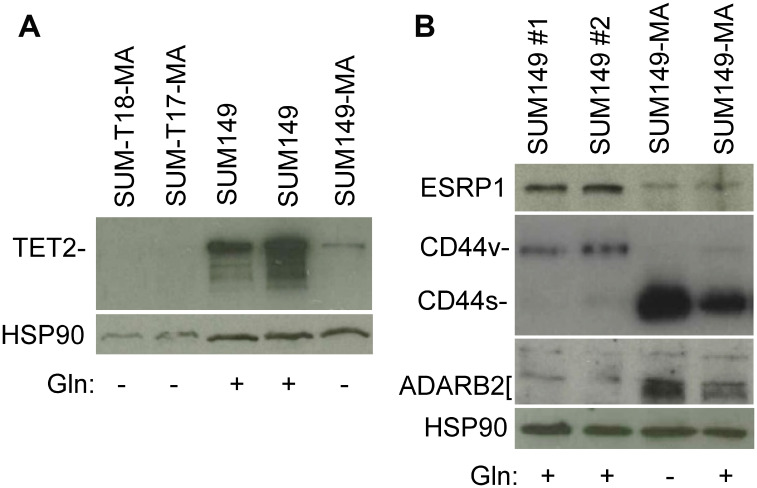
Validation of selected gene expression data with western blotting. (**A**) Relatively low level of TET2 protein in MA cells. Parental SUM149-Luc cells were cultured in glutamine (Gln)-containing medium with dialyzed fetal bovine serum (FBS; indicated in the figure as SUM149). SUM149-MA cells were grown in a glutamine-free medium with dialyzed FBS for 9 passages. SUM149-Luc cells were injected into the mammary fat pad of female nude mice, and the resulting tumors (SUM-T17 and SUM-T18) were mechanically disrupted and cultured directly in glutamine-free medium. This resulted in the growth of a few MA colonies, which were cultured in glutamine-free medium with dialyzed FBS for 3-4 passages. (**B**) Relatively low ESRP1 expression and high CD44s (at the expense of CD44v) and ADARB2 levels in MA cells. Parental SUM149-Luc cells were either grown in complete medium (SUM149 #1) or shifted to a glutamine-containing medium with dialyzed FBS for one passage (SUM149 #2). MA cells were cultured in glutamine-free medium with dialyzed FBS for 9 passages (Gln–) or were cultured without glutamine for 7 passages and then cultured in glutamine-containing medium for 2 passages (Gln+). Filters were re-probed with an HSP90 antibody to normalize sample loading.

TET2 is an α-ketoglutarate–dependent methylcytosine dioxygenase with important roles in regulating both the epigenome and transcriptome [17, 18]. TET2 mutations are one of the earliest genetic alterations in the evolution of acute myeloid leukemia and chronic myelomonocytic leukemia [19–21]. On the basis of our gene expression and CGH array data on SUM149-MA cells, which show *TET2* gene deletions and low *TET2* expression [12], we analyzed TET2 protein level in SUM149 parental and MA cells by western blotting. The TET2 protein level was >90% lower in MA cells than in the parental cells (Figure 2A). We observed a similarly dramatic decrease in TET2 levels in MA cells that were derived from xenograft tumors (SUM-T17-MA and SUM-T18-MA) generated by injecting mice with SUM149-Luc cells in the mammary fat pad and then cultured directly in glutamine-free medium (Figure 2A).

A low TET2 level in MA cells could be due to low gene dosage, but could also involve other mechanisms such as repression by ZEB1 as reported in glioma [22]; MA cells express a high level of ZEB1 as a part of their EMT program [12]. Although insufficient α-ketoglutarate cofactor could inhibit TET2, additional mechanisms such as repression of TET2 expression and *TET2* gene deletion (detected in MA cells) may be required to achieve a more durable low-TET2 progenitor-like state in MA cells similar to a therapy-resistant phenotype in TET2-mutant cancers [21]. Supporting the physiological relevance of this finding was our observation that the WT1 protein, whose interaction with TET2 governs its pattern of deposition on chromatin [23], is also downregulated in MA cells by gene deletion and repression, similar to TET2 [12]. These results are consistent with a prevailing model whereby epigenetic state could be influenced by availability of α-ketoglutarate, TET2, or WT1 in a mutually exclusive manner [23]. Our results suggest that a lack of glutamine (leading to a lack of α-ketoglutarate) would force the selection of an epigenetic state that does not require a high level of TET2, representing an example of how phenotypic selection for an adaptable metabolic state would dictate the selection of an interconnected overall adaptable embryo-like state.

### Transcriptome of SUM149-MA cells

MA cells possess a dramatically different transcriptional landscape than the parental cells, as illustrated by their gain of ZEB1 and loss of GRHL2 expression, as a part of a high-EMT, therapy-resistant state [12]. Our gene expression data showed that ESRP1, a major RNA splicing regulator in epithelial cells, is downregulated in MA cells [12]. Here, we validated that result by western blotting; ESRP1 expression was dramatically lower in MA cells than in parental cells (Figure 2B). One of the consequences of ESRP1’s effect on RNA splicing events is shifting of the balance between an mRNA variant encoding CD44v and another mRNA variant encoding CD44s; when ESRP1 is abundant, the balance shifts toward CD44v, and when ESRP1 is lacking, the balance shifts toward CD44s at the expense of CD44v [24, 25]. Our results, as anticipated, indicate a dramatic increase in CD44s and a corresponding decrease in CD44v in ESRP1-deficient MA cells [Figure 2B]. On the basis of this and findings by others, we conclude that MA cells express a high ZEB1/low ESRP1/high CD44s network that enforces self-renewal in cancer stem–like cells [24, 25].

Post-transcriptional modifications of RNA may offer unique opportunities for cell survival under extreme circumstances, e.g., a prolonged lack of glutamine, by re-purposing existing RNAs for survival-related functions. This line of thinking is supported by our observation of the involvement of FTO in initial colony growth of MA cells [13]. FTO, an α-ketoglutarate–dependent dioxygenase that demethylates m6A in RNAs to regulate energy balance, promotes organismal survival under conditions of nutritional scarcities [26, 27]. Furthermore, a reduction in TET2 level influences not only epigenome but transcriptome as well since it also modifies methylcytosine residues on RNA to hydroxymethylcytosine [18]. In addition, RNA-editing enzymes ADARB1 and ADARB2, which are involved in deamination of adenosine to inosine on RNAs, are overexpressed in MA cells; the *ADARB1* gene is also amplified (data in reference 12). Validation of the gene expression data with western blotting for ADARB2 showed a significantly higher level of ADARB2 protein in MA cells than in the parental cells (Figure 2B). The gene expression data indicate several other alterations in RNA modifications and RNA-binding proteins in SUM149-MA cells that we have not yet validated [12]. We propose that the cancer cells with a capacity to persist in reversible quiescence, e.g., highly resistant MA cells in our model and MRD in the body, may rely on such RNA modifications more than the proliferating cells.

### Evaluation of low-dose 6-mercaptopurine for efficacy on SUM149-MA cells

We chose to evaluate low-dose 6-MP mainly for its potential for re-purposing as a treatment for poor-prognosis MRD. Its successful clinical record in IBD over several decades indicates relative safety and provides ample clinical experience with its side effects [15]. 6-MP undergoes extensive metabolism after intake; its metabolites, thioguanine nucleotides, get mis-incorporated into RNA and DNA. The limited incorporation of thioguanine nucleotides into nucleic acids may affect the functionality and homeostasis of the abnormal T and B cells that are responsible for inflammatory bowel disease. Additional 6-MP effects may be due to the inhibition of Rac1 activation by mercapto-modified nucleotides in several cell types that normally relies on exchange of bound GDP with GTP [28]. Importantly, although it takes time (weeks to months) for 6-MP to relieve disease symptoms, it induces and maintains remission. These clinical findings indicate that 6-MP is able to control progenitor-like immune cells that drive the disease. Other medications that relieve symptoms in the acute phase of the disease by quickly inhibiting proliferating cells are ineffective in maintaining disease remission. In trying to repurpose 6-MP for controlling MRD in breast cancer, we recognize that breast cancer cells are different from immune cells. However, there are important similarities in the target cells and their microenvironment; the goal in both pathologies is to target progenitor-like cells that typically reside in a safe microenvironment in bone marrow. An additional rationale for choosing low-dose 6-MP was that it is a broadly active agent effective against heterogeneous disease, appropriate for the adaptable embryo-like SUM149-MA cells that can enter reversible quiescence to survive difficult challenges and proliferate when conditions are favorable.

RNA processing plays an important role in cellular adaptability to complement other mechanisms such as embryo-like state and genetic alterations affecting cell cycle checkpoints and genomic instability. Therefore, mis-incorporation of 6-MP metabolites into RNAs could potentially disrupt RNA structure and affect RNA processing occurring via several mechanisms, thus impeding the MA cells’ adaptability and capacity to generate diversity [29–31]. In a survey of selected compounds that might inhibit MA cells, we had found 6-MP to be efficacious [12]. Here we conducted a more detailed investigation into the nature of response and resistance upon 6-MP treatment to address some important issues that are relevant for the clinic.

**Figure 3:**
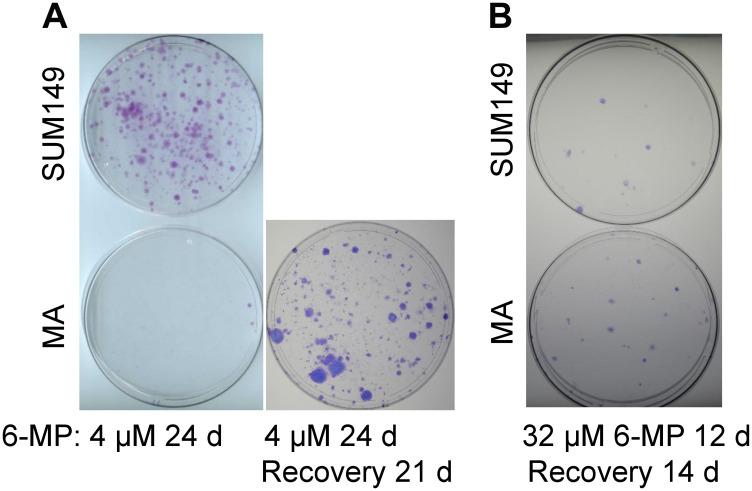
Differential effects of low-dose versus high-dose 6-MP on MA cells. (**A**) Low-dose 6-MP inhibited MA cells to a greater extent than it inhibited parental SUM149 cells. Duplicate dishes similar to those shown in Supplementary Figure 1 that reached confluency after treatment with 4 μM 6-MP for 7 days were passaged at a 1:10 ratio while continuing treatment with 6-MP. The dishes were stained with crystal violet after a total 24 days treatment with 6-MP. MA cells in a duplicate dish were allowed to recover without 6-MP for 21 days and form colonies. Representative cell cultures are shown. (**B**) The effects of high-dose 6-MP treatment in SUM149 cells were not significantly different than the effects in SUM149-MA cells. MA or parental cells were treated with 32 μM 6-MP for 6 days and passaged at a 1:10 ratio; 6-MP treatment continued for another 6 days. This 6-MP treatment killed >99% cells in the dishes, as indicated by microscopic examination. 6-MP was removed and surviving cells were cultured for 14 days, when colonies were stained. Representative cell cultures are shown.

We treated SUM149-MA cells and parental SUM149 cells, both growing in a medium containing glutamine, with relatively low doses of 6-MP. 6-MP did not significantly affect the growth of either type of cells at concentrations up to 8 μM for 7 days (Supplementary Figure 1). Cells in all dishes reached confluency at the same time as vehicle-treated controls. When we passaged the cell cultures while continuing to treat with 6-MP, however, we noticed significant growth inhibition in both types of cells (Figure 3A). Treatment with 4 μM 6-MP for 24 days inhibited the growth of SUM149-MA cells very dramatically; only a couple of colonies were observed on the dish (Figure 3A). Although the growth of the parental cells was affected, the degree of inhibition was significantly less than that observed in MA cells. This result is the opposite of results obtained with cell proliferation inhibitors, including chemotherapeutic drugs; MA cells are more resistant than parental cells to such treatments [12]. We postulate that the greater 6-MP sensitivity in MA cells could be related to its capacity to interfere with transcriptome modifications that these cells rely upon.

Next, to determine whether the 4 μM 6-MP treatment had killed or irreversibly disabled MA cells or only arrested them in reversible quiescence, we removed the drug from duplicate dishes treated in parallel with the ones represented in Figure 3A and allowed the cells in these dishes to recover in drug-free medium for 21 days. A significant number of colonies appeared in the drug-free dishes, indicating that 6-MP inhibited the growth of colonies but did not eradicate “progenitor-like” resistant cells that grow into colonies (Figure 3A). Instead, 6-MP arrested such cells in reversible quiescence. This result is not surprising in light of the high intrinsic resistance of MA cells—it also parallels the effect of 6-MP treatment in IBD—controlling the cell number and activation status of rogue immune cells, but not eradicating the abnormal progenitor cells. Interpreted in the context of poor-prognosis MRD, these results imply that 6-MP could potentially prolong the dormancy of MRD, thus improving outcomes.

### Evaluation of a chemotherapy-like high-dose 6-MP

6-MP at higher doses is used in cancer chemotherapy, in combination with methotrexate for treating acute lymphoblastic leukemia; it kills proliferative cancer cells by damaging RNA and DNA in this setting [32]. To determine the effect of a high chemotherapy-like dose on MA cells, we treated the cells with 32 μM 6-MP for 12 days (sufficient to kill more than 99% cells as determined by microscopy), and then allowed them to recover for 14 days in a drug-free medium. MA cells treated this way did not yield fewer colonies than the parental cells; in fact, they produced slightly more colonies than the parental cells (Figure 3B). More data pertaining to treatment with low dose (2–8 μM range) and high dose (32–16 μM range) 6-MP are shown in Supplementary Figure 2. These results are consistent with our hypothesis that a low dose 6-MP used for a long time would be more effective than a high-dose 6-MP used for a short time on highly adaptable MA cells-like cancer cells. Above all, these results illustrate the usefulness of our cell culture-based model of highly adaptable cancer cells in evaluating non-cytotoxic therapies that could be offered at a more treatable stage of evolving MRD, rather than at metastasis.

### 6-MP treatment does not increase resistance in 6-MP-resistant cells

**Figure 4:**
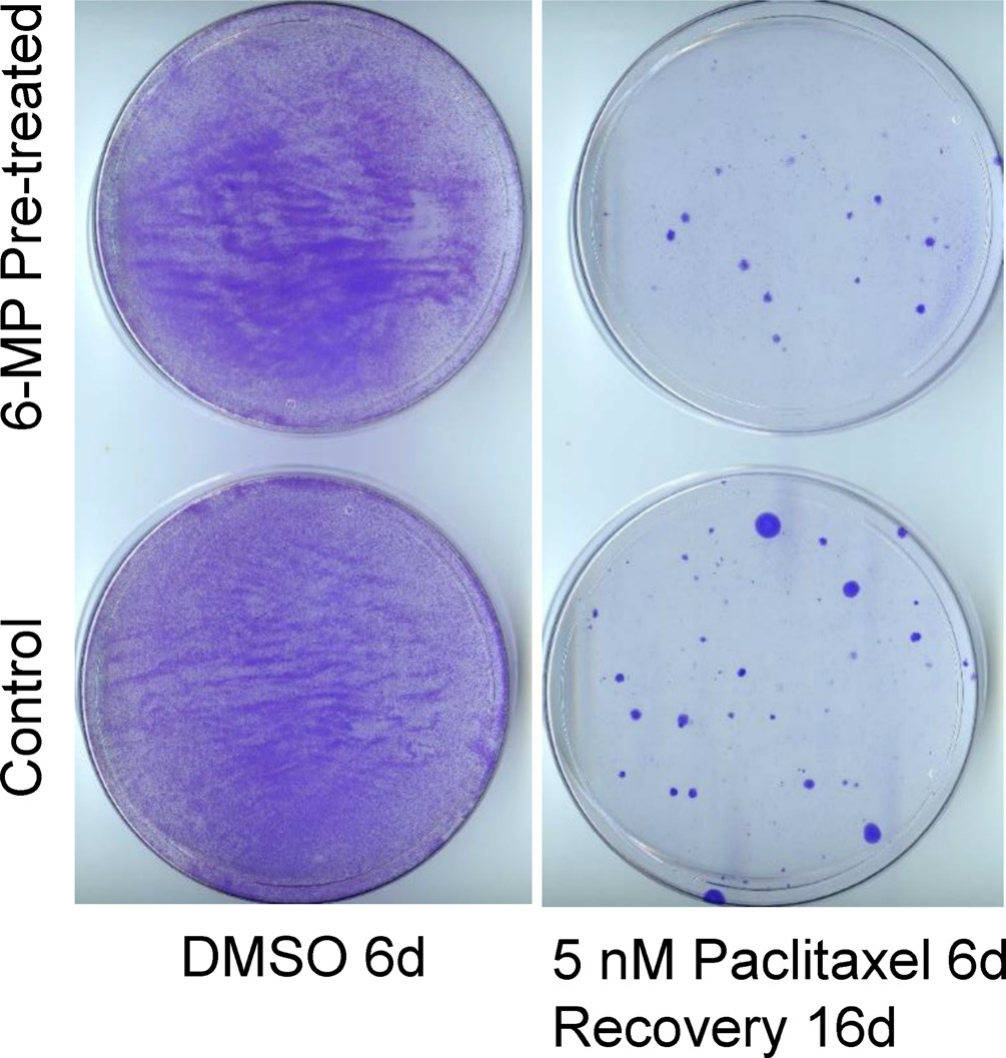
SUM149 cells surviving 6-MP treatment do not have increased resistance to paclitaxel. SUM149-Luc cells were pretreated with 8 μM 6-MP for 24 days, then were allowed to recover and grow without 6-MP for 12 days. The 6-MP-pretreated and -untreated control cells were treated with 5 nM paclitaxel for 6 days (which killed >99% of cells), then allowed to recover and form colonies for 16 days, when they were stained. Cells treated with dimethyl sulfoxide (DMSO) solvent in parallel served as controls. Representative cell cultures are shown.

Since low-dose 6-MP was less effective on proliferating cells than on MA cells, one important issue to address would be whether 6-MP treatment renders non-responding cancer cells resistant to other therapies. We treated SUM149 cells with 6-MP for 28 days, passaged the resulting colonies for growth in the absence of 6-MP for 12 days, and then tested their response to paclitaxel. We found that the numbers of colonies growing in cell cultures pretreated with 6-MP and then treated for 6 days with 5 nM or 10 nM paclitaxel were comparable to those in cell cultures not pretreated with 6-MP (compare panels in Figure 4). Although the use of chemotherapeutic drugs may not be a reasonable option at the MRD stage, an evaluation like this may be informative in thinking about response versus resistance upon treatment with 6-MP. Response to chemotherapeutic drugs *in vitro* could be used as a surrogate for the body’s responses against cancer; in other words, the type of cells that are sensitive to chemotherapeutic drugs may also be sensitive to killing by other means, e.g., by immune system.

### Low-dose 6-MP inhibits FC-IBC02 and FC-IBC02-MA cells

**Figure 5:**
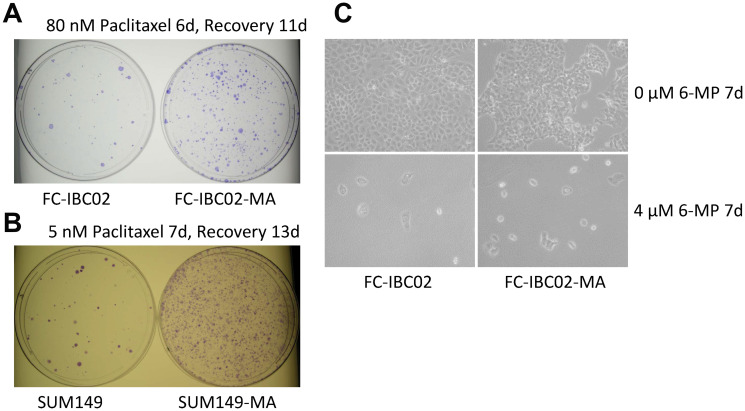
6-MP severely inhibits highly resistant FC-IBC02 and FC-IBC02-MA cells. (**A**) High resistance to paclitaxel in FC-IBC02 and FC-IBC02-MA TNBC cells. The cells were plated (1 × 10^6^ cells per dish) in Ham F12 medium with glutamine, treated beginning the next day with 80 nM paclitaxel for 6 days, then allowed to recover and grow into colonies for 11 days. Since FC-IBC02 cells were originally cultured in Dulbecco modified Eagle medium (DMEM), we selected MA cells in glutamine-free DMEM, then adapted them to the Ham F12 medium, first without glutamine and finally with glutamine (to match the medium used for SUM149 cells for comparison). Parental cells were also adapted to Ham F12 medium for the experiment. (**B**) Paclitaxel resistance in SUM149-MA cells. SUM149 and SUM149-MA cells were treated with 5 nM paclitaxel for 7 days, then allowed to recover and grow into colonies for 13 days before staining. (**C**) High sensitivity to 6-MP in FC-IBC02 cells. FC-IBC02 and FC-IBC02-MA cells were plated (5 × 10^5^ cells per dish) in DMEM with glutamine and immediately treated with 4 μM 6-MP or with 0 μM 6-MP for 7 days. Representative images taken at 10 × magnification are shown.

FC-IBC02, another TNBC cell line, was established from a pleural effusion of a patient with very resistant inflammatory breast cancer [33]. We recently reported that glutamine deprivation in this cell line selected rare metabolically adaptable cancer cells termed FC-IBC02-MA, which are resistant to doxorubicin, like SUM149-MA cells [14]. The FC-IBC02 cell line is highly resistant to paclitaxel, requiring 80 nM paclitaxel for the degree of inhibition that is achieved in SUM149 cells by 5 nM paclitaxel (compare Figure 5A and 5B). Similar to the SUM149-MA cells being more paclitaxel-resistant than SUM149 cells, FC-IBC02-MA cells were more resistant to paclitaxel than the parental cells, yielding approximately 5–10 times more colonies than parental cells after treatment for 6 days (Figure 5A). Surprisingly, treatment for 7 days with low-dose (4 μM) 6-MP markedly inhibited growth and induced cell killing in both FC-IBC02-MA and the parental cells (Figure 5C). This inhibition was more rapid and severe than that in SUM149-MA and SUM149 cells (compare Figure 3A and Figure 5C).

To make the FC-IBC02-MA cell model relevant to an MRD setting, we first determined the cells’ response to lower doses of 6-MP for long periods of treatment. Treatment for 30 days with 0.5 μM 6-MP eliminated all FC-IBC02-MA cells, since no colonies appeared after a subsequent 31 days recovery (Supplementary Figure 3). A parallel 30 days treatment with 0.25 μM 6-MP followed by a 31-day recovery yielded one colony, while a similar treatment with 0.125 μM 6-MP followed by a 31-day recovery yielded approximately 100–200 colonies (Supplementary Figure 3). Upon even longer treatment (61 days), 0.25 μM or higher doses of 6-MP maintained complete inhibition of clonogenic growth. Treatment with the lowest dose (0.125 μM) of 6-MP for 61 days resulted in a couple of colonies plus a few very small colonies (Supplementary Figure 3). Thus our results support the hypothesis that reprogramming occurring in breast cancer cells that drive cancer evolution under selective pressures of multiple therapies could render such cells sensitive to inhibitors of reprogramming such as 6-MP.

### Evaluation of a combination therapy with 6-MP

**Figure 6:**
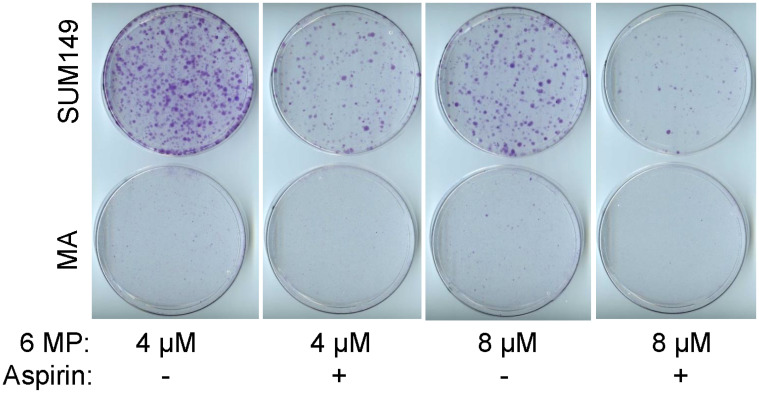
The combinatorial effect of aspirin with 6-MP. SUM149-Luc and MA cells were treated with 4–8 μM 6-MP alone or in combination with 0.625 mM aspirin for 21 days. The cells were passaged once at a 1:10 ratio during the treatment: parental SUM149-Luc cells on day 6 and MA cells on day 8. The dishes were stained with crystal violet.

Considering the high degree of resistance to therapy in MA cells, a good application of our cell culture model may be in evaluating combination therapies. We evaluated a combination of 6-MP with aspirin at a relatively low dose that did not have a significant impact on cell proliferation. Aspirin is utilized as an inhibitor of cyclooxygenase 1 and cyclooxygenase 2; both these proteins may be upregulated in MA cells as indicated by our gene expression microarray data [12]. We chose aspirin mainly for its safety profile and for its potential to inhibit proteome reprogramming through acetylation [34], analogous to 6-MP’s potential to interfere with transcriptome reprogramming. After evaluating several doses, we chose a relatively low dose of aspirin that does not significantly inhibit cell proliferation as compared to vehicle-treated cells. The combinatorial effect of aspirin with 6-MP in SUM149 cells is illustrated by our finding that cells treated with the combination formed fewer colonies than those treated with 6-MP alone; colony formation was inhibited by more than 50% by 0.625 mM aspirin with either 4 μM or 8 μM 6-MP (Figure 6). Although 6-MP alone inhibited colony growth of SUM149-MA cells, as described above, growth was further inhibited by 0.625 mM aspirin, as indicated by decreases in both the rare small colonies and tiny colonies (compare dishes in Figure 6). Although this experiment shows the value of our model for evaluating combination therapies, this specific combination therapy may not be optimal at the MRD stage. We used the aspirin doses that are in the lower range of those used in cell culture, they are still not achievable in the body. To be an optimist, if the effects of a low-dose aspirin were cumulative over time, it could prove to be useful for halting the progression of poor prognosis MRD to clinical metastasis. At present, we must find better agents and better combinations that would go further toward overcoming resistance in MA cells than 6-MP alone.

**Figure 7:**
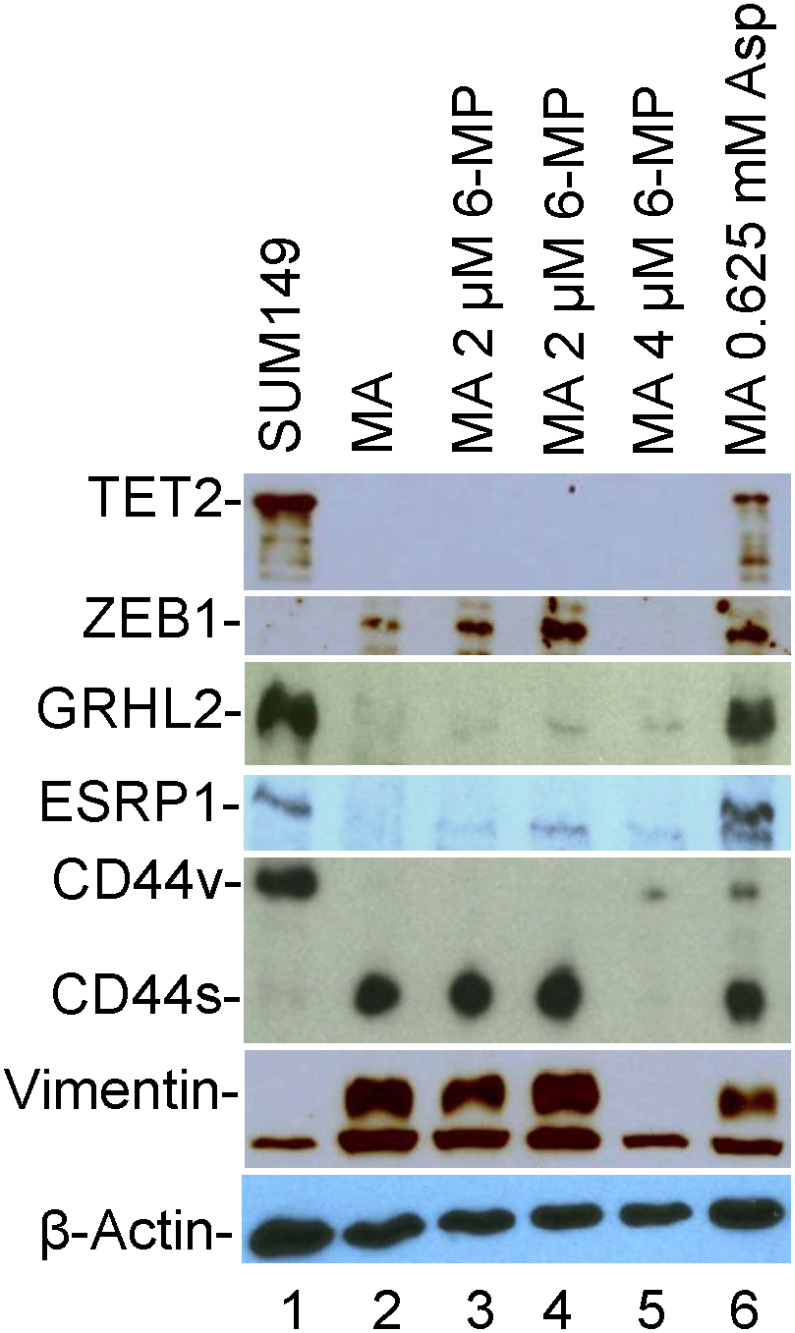
The effects of 6-MP and aspirin on TET2 and EMT-related proteins in SUM149-MA cells. We detected the indicated proteins by western blotting. Lane 1: luciferase-transfected parental cell line SUM149. Lane 2: MA cells selected from the SUM149-Luc cell line. Lanes 3 and 4: MA cells treated with 2 μM 6-MP for 32 days followed by a recovery in a drug-free medium for 8 days (Lane 3) or for 26 days (Lane 4). Lane 5: MA cells treated with 4 μM 6-MP for 24 days followed by a recovery in a drug-free medium for 34 days. Lane 6: MA cells treated with 0.625 mM aspirin for 49 days. The β-actin blot is a re-probe of the vimentin blot.

To determine whether the long treatments with 6-MP and aspirin affect the EMT in MA cells, we analyzed some important indicators of EMT by western blotting. We found that while 2 μM 6-MP did not affect any of the EMT markers, 4 μM 6-MP affected some of the markers- it dramatically reduced EMT markers ZEB1, CD44s (with appearance of CD44v), and mesenchymal marker vimentin (compare lanes 2 and 5 in Figure 7). However, it did not increase the levels of epithelial markers GRHL2, ESRP1, or “differentiation” indicator TET2 (Figure 7). Treatment with o.625 mM aspirin resulted in an increase in TET2, GRHL2, ESRP1, and CD44v, and a modest 50% decrease in vimentin (compare lanes 2 and 6 in Figure 7). All these results indicate that aspirin treatment modifies the cells towards an epithelial state; since all the EMT markers (ZEB1, ESRP1, CD44s) are still present after aspirin treatment, it is likely that reversal of EMT occurs in some but not all MA cells.

## DISCUSSION

Our experimental design allows the phenotypic selection of those rare cancer cells that can survive in quiescence, adapt over a few weeks, and then manage to exit quiescence as indicated by growth of colonies. A severe metabolic challenge such as the one used for selecting MA cells selects for adaptability that could be governed by both genetic and epigenetic means. It is possible to imagine different mechanisms of adaptation operating at different time scales in a cooperative manner. While RNA modifications (some reversible, others irreversible) could be useful for dynamic regulation relatively quickly, genetic alterations would be better suited for a relatively stable response.

Our results obtained with highly resistant SUM149-MA and FC-IBC02-MA cells indicate that low-dose 6-MP may be useful for halting progression of poor-prognosis MRD to metastasis in TNBC patients. Our results, along with the results obtained with 6-MP for treating IBD, suggest that a low dose of 6-MP will not eradicate the deepest roots of resistance in the form of progenitor-like cancer cells (which produced the rest of the MA cells in our study) that can survive in reversible quiescence (Figure 3). It will, however, inhibit the MA cells as they initiate cell proliferation (Figure 3). These results indicate that 6-MP may affect the structure and reprogramming of RNAs (both non-coding and coding); the highly adaptable subpopulation of cancer cells would be more sensitive than other cancer cells because of their high reliance on reprogramming of transcriptome. Thus 6-MP may inhibit RNA-based cell regulation that may be particularly important in heterogeneous cancers such as TNBC [29, 31, 35].

The main purpose of this study was to illustrate how therapies could be chosen and evaluated for their efficacy against high adaptable and heterogeneous cancer in cell culture, rather than to prove that 6-MP treatment would eradicate or disable all adaptable cancer cells. Cancer cell lines are generally viewed as poor models of intra-tumor heterogeneity. Our work provides strong evidence that a rare subpopulation of highly adaptable cancer cells in TNBC cell lines, which can be selected under a metabolic bottleneck, is capable of generating a tremendous cellular heterogeneity in genome, epigenome, transcriptome, proteome, and so on [12]. Based on significant clinical data from the success of 6-MP in treating IBD, and based on our data that: 1) long treatment with low-dose 6-MP is more effective on MA cells than short treatment with high-dose 6-MP; 2) the effect of 6-MP is cumulative over time; and 3) there is a low probability of development of resistance to 6-MP, a case could be made for re-purposing 6-MP for treating poor-prognosis MRD in TNBC. Promising early results in a phase II clinical trial investigating whether 6-MP might be an effective treatment for BRCA-deficient tumors even after the development of resistance to PARP inhibitors or platinum drugs provide further support to this idea [36].

It is important to recognize that even if 6-MP treatment of poor-prognosis MRD improves the recurrence-free survival rate, there will be resistance to therapy. Based on experience in treating IBD, one mechanism behind 6-MP’s lower efficacy in some patients involves its conversion to inactive 6-thiouric acid, catalyzed by xanthine oxidase, which can be addressed by co-therapy with allopurinol [37]. Other mechanisms that may reduce 6-MP efficacy in cancer include, for example, transcriptome reprogramming through alternative paths. We could investigate whether intermittent therapy with two different nucleoside analogs is better than a single drug.

## MATERIALS AND METHODS

### Cell lines and culture

The well-characterized TN-IBC cell line SUM149 (sumlineknowledgebase.com), originally obtained from Stephen Ethier (Barbara Ann Karmanos Cancer Institute, Detroit, MI, USA), was grown in Ham F-12 medium supplemented with 5% fetal bovine serum (FBS), 5 μg/mL insulin, 1 μg/mL hydrocortisone, 100 U/mL penicillin, and 100 μg/mL streptomycin in a humidified 5% CO_2_ atmosphere. We previously described SUM149-Luc, the luciferase-transfected SUM149 cells [38]. The relatively new and less characterized TN-IBC cell line FC-IBC02, established from a pleural effusion from a patient with a secondary IBC, was originally developed by Dr. Massimo Cristofanilli [33] and was obtained from the cell line collection of the Morgan Welch Inflammatory Breast Cancer Research Program and Clinic at MD Anderson Cancer Center. It was grown as an adherent culture in Dulbecco modified Eagle medium (DMEM) supplemented with 10% FBS. The cell lines were authenticated by short tandem repeat analysis at our institutional core facility.

We previously described the selection and growth of rare MA cells from SUM149-Luc and FC-IBC02 cell lines in glutamine-free medium containing dialyzed FBS, which has a very low level of glutamine (Thermo Fisher Scientific, Waltham, MA, USA) [11, 12, 14]. The MA cells can be passaged indefinitely in glutamine-free medium with dialyzed FBS. However, to minimize the loss of cellular characteristics in cell culture, we used MA cells that were in a glutamine-free medium for fewer than 10 passages. Similarly, for investigating the behavior of MA cells in a glutamine-containing medium, we used cells cultured for fewer than 10 passages.

### Western blotting

We performed western blotting as described previously [39]. The following primary antibodies were used for protein detection: anti-TET2 (catalog number 61390, Active Motif, Carlsbad, CA), anti-ESRP1 (catalog number GTX131373, GeneTex, Irvine, CA, USA), anti-CD44 (catalog number MAB7045, R&D Systems, Minneapolis, MN, USA), anti-ADARB2 (catalog number sc-73410, Santa Cruz Biotechnology, Dallas, TX, USA), anti-ZEB1 (catalog number 3396, Cell Signaling Technology, Danvers, MA, USA), anti-GRHL2 (catalog number HPA004820, Sigma-Aldrich, St. Louis, MO), and anti-vimentin (catalog number 3932, Cell Signaling Technology). The ECL prime blocking agent (GE Healthcare Life Sciences, Piscataway, NJ, USA) was used for blocking and Lumigen TMA-6 reagents for detection (Lumigen, Inc., Southfield, MI, USA). The blots were re-probed with an HSP90 antibody (catalog number 4875, Cell Signaling Technology) or with an anti-β-actin antibody (catalog number A5316, Sigma-Aldrich) to document equal protein loading. Each western blot was performed at least twice; representative blots are shown. Relative intensities of the bands were determined with ImageJ software (National Institutes of Health, Bethesda, MD, USA).

### Reagents and drugs

The drugs used in this study (6-MP, paclitaxel, and aspirin) were all purchased from Sigma-Aldrich (St. Louis, MO, USA). 6-MP was dissolved in 0.1 M NaOH, paclitaxel in dimethyl sulfoxide (DMSO), and aspirin in ethanol. Equal volumes of the solvents without drugs were added to the control dishes. Solvent volume was 0.04% of the volume of the culture medium, excepting ethanol (solvent for aspirin), which was added to 0.25% of the volume of the culture medium.

### Assay of relative resistance in MA cells

To investigate the efficacy of potential therapeutic agents on highly resistant MA cells, which are in equilibrium with a large percentage of proliferating relatively sensitive cells, we treated MA and parental cells in parallel for long periods. We followed up to determine whether the non-proliferating cells remaining after such treatments were capable of forming colonies upon removing the drug. To determine whether long treatment with a drug such as 6-MP affected the sensitivity of cells to chemotherapeutic drugs, we first allowed drug-treated cells to recover for a few days and then passaged. We treated these drug-treated cells in parallel with the control vehicle-treated cells for 6–7 days with pre-determined concentrations of chemotherapeutic drugs expected to kill 99% of proliferating cells. We then removed the chemotherapeutic drugs and allowed surviving cells to form colonies for 2–4 weeks. Colonies were stained with crystal violet.

### Evaluation of 6-MP in cell culture

To evaluate the effect of low-dose 6-MP on parental and MA cells, we plated 5 × 10^6^ cells per 10-cm dish in duplicate in culture medium with glutamine containing dialyzed FBS. 6-MP was added the next day, and the resulting cultures were examined under a microscope at least twice a week and representative areas photographed to document the effect of the treatment. We also stained the dishes with crystal violet when appropriate and photographed or scanned them. Low-dose 6-MP did not have a significant growth-inhibitory effect for 7 days; therefore, cultures were passaged at the usual 1:10 ratio and 6-MP treatment was continued for another 17 days before the dishes were stained with crystal violet. We evaluated high-dose 6-MP similarly except that the treatment time was shorter to accommodate faster cell killing at high doses.

### Assay of relative resistance to chemotherapeutic drug paclitaxel

Parental FC-IBC02 and FC-IBC02-MA cells were plated in 10-cm dishes with culture medium containing glutamine. After 24 hours, 80 nM paclitaxel was added. Drug treatment continued for 6–7 days while response to the drug was monitored under a microscope; this treatment typically killed the majority of cells, leaving behind only the most resistant cancer cells. The drug-containing medium was removed, and the cells were washed twice with phosphate-buffered saline solution and incubated in the glutamine-containing medium without drug for several days to weeks until colonies visible to the naked eye appeared. The colonies were stained with crystal violet and photographed or scanned. We used a similar method to evaluate relative resistance to paclitaxel in SUM149-Luc cells treated with 6-MP or untreated, except that the paclitaxel dose was in the 5–10 nM range.

## SUPPLEMENTARY MATERIALS



## Abbreviations

EMT: epithelial to mesenchymal transition
DMEM: Dulbecco modified Eagle medium
DMSO: dimethyl sulfoxide
FBS: fetal bovine serum
IBC: inflammatory breast cancer
MA: metabolically adaptable
MRD: minimal residual disease
TNBC: triple-negative breast cancer
TN-IBC: triple-negative inflammatory breast cancer
6-MP: 6-mercaptopurine
