# Correction: Evaluation of 6-mercaptopurine in a cell culture model of adaptable triple-negative breast cancer with metastatic potential

**DOI:** 10.18632/oncotarget.28311

**Published:** 2022-11-17

**Authors:** Balraj Singh, Vanessa N. Sarli, Hannah E. Kinne, Anna Shamsnia, Anthony Lucci

**Affiliations:** ^1^Department of Breast Surgical Oncology, The University of Texas MD Anderson Cancer Center, Houston, TX, USA; ^2^Morgan Welch Inflammatory Breast Cancer Research Program and Clinic, The University of Texas MD Anderson Cancer Center, Houston, TX, USA

**This article has been corrected:** Figure 1 contains two accidental duplicate images representing MA cells growing without and with glutamine, respectively. The corrected Figure 1, obtained with independently collected new images in the laboratory, is shown below. The authors declare that these corrections do not change the results or conclusions of this paper.

Original article: Oncotarget. 2019; 10:3681–3693. 3681-3693. https://doi.org/10.18632/oncotarget.26978

**Figure 1:**
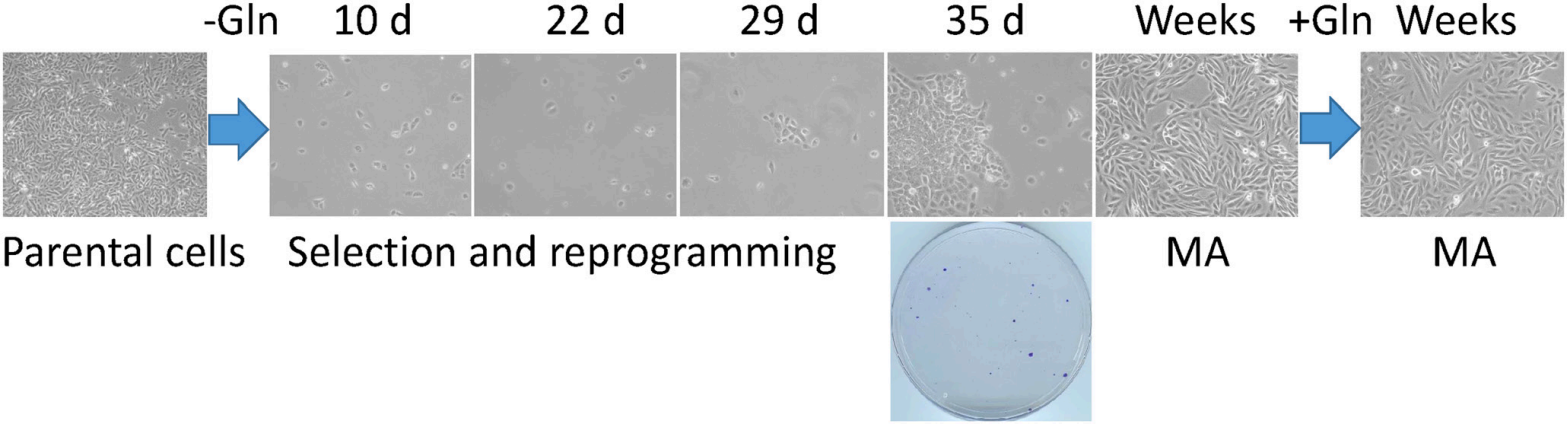
A cell culture model of the rare cancer cells that survive a severe metabolic challenge and “evolve” to emerge as highly adaptable. Triple-negative breast cancer SUM149-Luc cells were plated in 10-cm dishes (5 × 10^5^ per dish) in culture medium containing dialyzed FBS and no glutamine (Gln). While >99.9% of the cells died quickly, a small number of cells survived in quiescence for 3–4 weeks; there were innumerable abortive attempts at cell growth during this period. We postulate that a few cells in this initial period of 3–4 weeks “evolved” to a point that they eventually succeeded in forming colonies. Shown are representative cell cultures (10 × magnification) at various stages, along with a stained dish at 5 weeks (representative image taken from data in reference 13). Metabolically adaptable (MA) cancer cells selected in this manner can be cultured indefinitely in a medium without or with glutamine; representative MA cultures depicting mesenchymal morphology in both media are shown.

