# Supplementary material for: Evaluation of 6-mercaptopurine in a cell culture model of adaptable triple-negative breast cancer with metastatic potential
Source: Oncotarget. 2019 Jun 4;10(38):3681–93. doi: 10.18632/oncotarget.26978 (PMC6557209; doi:10.18632/oncotarget.26978)
Supplement: Supplementary file 1 [file oncotarget-10-3681-s001.pdf]

Evaluation of 6-mercaptopurine in a cell culture model of adaptable triple-negative breast cancer with metastatic potential

SUPPLEMENTARY MATERIALS

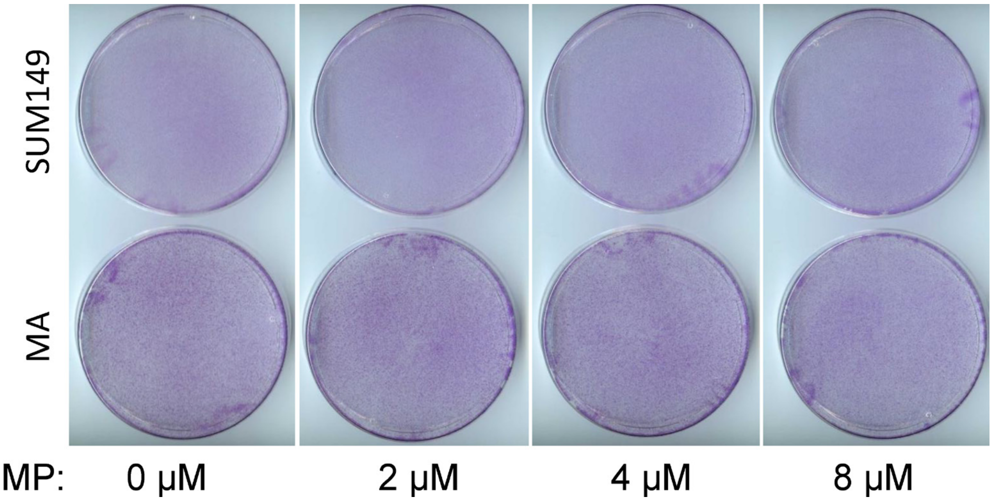

Supplementary Figure 1: A low dose of 6-MP does not significantly inhibit cell proliferation in 7 days (related to Figure 3). Parental SUM149-Luc or metabolically adaptable (MA) cells were plated on 10 cm dishes and treated with 2–8 µM 6-MP, beginning the next day, for 7 days. Cells in all dishes reached confluency to an extent similar to that of control dishes. The dishes were stained with crystal violet.

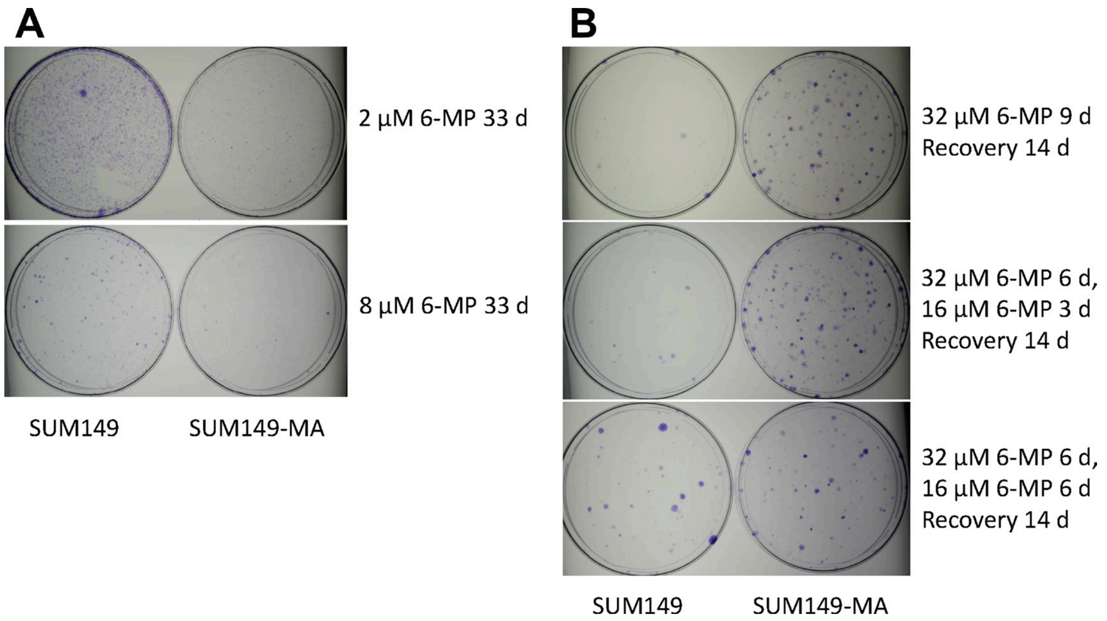

Supplementary Figure 2: Differential effects of low-dose versus high-dose 6-MP on MA cells (related to Figure 3). (**A**) Long treatments with low-doses of 6-MP inhibited MA cells to a greater extent than it inhibited parental SUM149 cells. Cells treated with 2 μM 6-MP were passaged at day 10 and at day 16 during a total treatment for 33 days. Cells treated with 8 μM 6-MP were passaged at day 9 during a total treatment for 33 days. All dishes were stained after a 33 days treatment. (**B**) Effect of high-dose 6-MP. MA or parental cells were treated with 32 μM 6-MP for 6 days and passaged at a 1:10 ratio. 6-MP treatment continued with a 32 μM dose for another 3 days (top), or with a 16 μM dose for 3 days (middle) or 6 days (bottom). This 6-MP treatment killed >99% cells in the dishes, as indicated by microscopic examination. 6-MP was removed and surviving cells were cultured for 14 days, when colonies were stained. Representative cell cultures are shown.

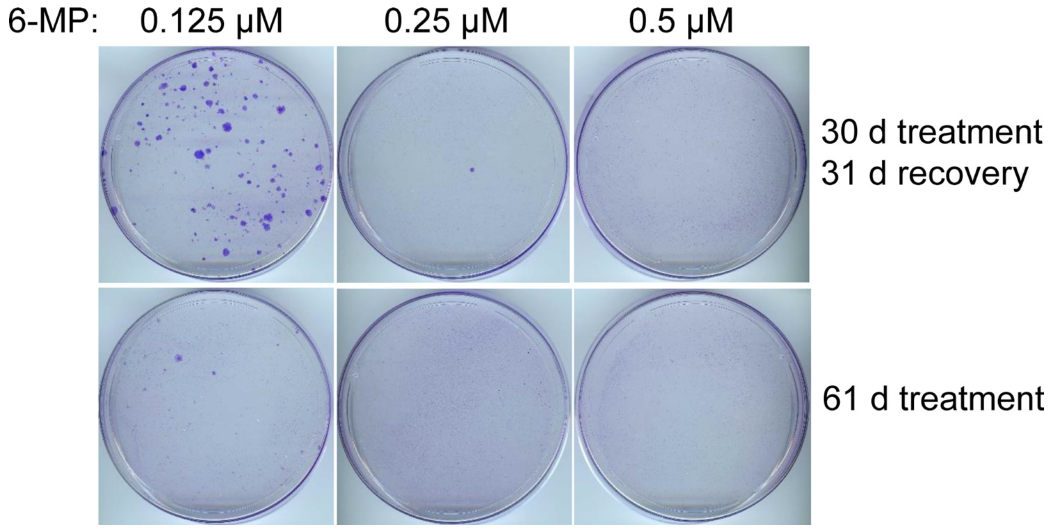

Supplementary Figure 3: Continued inhibition of FC-IBC02-MA cells with low doses of 6-MP (related to Figure 5). Cells were treated with indicated doses of 6-MP for 30 days followed by recovery in a drug-free medium for 31 days (top). Parallel dishes were treated with 6-MP for 61 days (bottom). The dishes were stained with crystal violet.
